# Can Regional Integration Reduce Urban Carbon Emission? An Empirical Study Based on the Yangtze River Delta, China

**DOI:** 10.3390/ijerph20021395

**Published:** 2023-01-12

**Authors:** Dongsheng Yan, Pingxing Li

**Affiliations:** 1 Public Administration School, Hohai University, Nanjing 211100, China; 2 Key Laboratory of Watershed Geographic Sciences, Nanjing Institute of Geography and Limnology, CAS, Nanjing 210008, China

**Keywords:** carbon emission, effect, mechanism, quasi-natural experiment, regional integration, the Yangtze River Delta

## Abstract

Regional integration can significantly affect carbon emissions, but scholars have paid more attention to the impact of integration level, ignoring the importance of regional integration expansion. This study attempts to demonstrate whether, in the process of promoting carbon peak and carbon neutrality in China, the transformation of the administrative region’s economy into an integrated economy based on urban agglomeration regional integration expansion affects urban carbon emissions. This study considers the regional integration expansion of the Yangtze River Delta Urban Economic Coordination Committee as a quasi-natural experiment, exploring the carbon emission reduction effect of regional integration with the difference-in-differences model. With the mediating and moderating effect models, this study examines the mechanism of regional integration affecting urban carbon emissions. The results show that regional integration, considering regional integration expansion, can significantly reduce urban carbon emissions. The carbon emission reduction effects of regional integration show significant heterogeneity. For example, there is a significant carbon emission reduction effect of high-hierarchy cities and an insignificant carbon emission reduction effect of general-hierarchy cities. Further research into the driving mechanism finds that deepening collaborative governance, industrial structure optimization, and green technology promotion brought about by regional integration are important mechanisms influencing urban carbon emissions. In addition, the carbon emission reduction effect of regional integration is influenced by the level of urban marketization and development efficiency. Different from the existing studies focusing on the effects of regional integration level, this study assesses the feasibility of promoting urban green development through urban agglomeration regional integration expansion. Based on the relevant empirical research, we propose to better promote high-quality development by strengthening urban agglomeration cooperation, optimizing urban development paths, strengthening innovative development, and improving macro political systems. It also indicates that the relevant policies should be formulated after considering local conditions.

## 1. Introduction

During rapid global economic development, the explosive growth of greenhouse gases (mainly CO_2_), resulting in global warming and increasingly frequent extreme climate crises, have raised huge challenges to global sustainable development [1]. To cope with this global environmental shock, promoting carbon emission reduction during economic growth has become the mainstream scientific consensus and focus of global attention [2,3]. Specifically, since the Kyoto Protocol came into force in 2005, low-carbon development has become a realistic path for all countries worldwide. China has grown rapidly to become the world’s second-largest economy, but its carbon emissions during economic growth are also among the highest worldwide [3]. For China, the development environment has deteriorated significantly, meaning that the factor-driven economic growth model with large-scale carbon emissions is difficult to sustain [3,4,5]. Chinese President Xi Jinping announced China’s carbon peak and carbon neutrality target at the 75th session of the United Nations General Assembly, which means that reducing carbon emissions will be a key initiative to promote high-quality development. It embodies a great power’s responsibility toward global carbon reduction action.

Scholars have conducted extensive research on related issues, and mainly focus on carbon emission measurement, spatial pattern analysis, and driver identification. (1) Scholars have measured total carbon emissions [5], and the carbon emissions of different industries [2,6,7] based on either (i) the energy consumption scale, (ii) the IPCC carbon emission estimation formula [2], or (iii) the night light inversion method [1]. They found that cities consume 60%–80% of energy and emit 70% of carbon emissions [5]. (2) The spatial evolution characteristics of carbon emissions were analyzed using variance coefficient, kernel density analysis, and spatial autocorrelation [4,8]. For example, the carbon emission intensity of Chinese cities shows a decreasing trend, which is more prominent in the south-central and eastern cities [8]. (3) Scientifically identifying the influencing factors of carbon emissions is also an important theme [9,10]. Based on the multivariate analysis method, it was found that economic development, industrial structure, infrastructure, technology progress, internationalization, and macro policies are all important factors affecting carbon emissions [2,3,9,10,11,12]. For example, the existence of an environmental Kuznets curve has been verified in the field of economic development [11]. The process of rapid and uncontrolled urbanization has a strong relation with growing carbon emission in Mexico [13]. In the area of openness, there is controversy over the environmental effects of foreign investment as a pollution halo or pollution paradise [14,15]. In terms of marketization, an increasing number of scholars have realized that segmented markets are not conducive to reducing carbon emissions [16,17]. For instance, EU integration and urban agglomeration integration in China have significantly reduced carbon emissions [3,18]. Therefore, many scholars have proposed promoting carbon emission reductions by promoting regional market integration [19].

The promotion effect of EU integration on carbon emission reduction has triggered relevant discussions [20]. For China, the transformation of administrative economies into integrated economies continues to deepen local government cooperation. A real need and consensus have emerged around urban agglomeration as an integrated spatial carrier that can be used to achieve green development [3,18,21]. In the integration process led by the well-functioning government of China, an increasing number of cities expect to participate in integration to achieve higher-quality development. Expansion will then become the norm of regional integration development [22]. Scholars have conducted many studies on integration effects, but several issues deserve further exploration. (1) Studies have demonstrated that regional integration can significantly promote green development [3,16,18]. However, scholars have mainly researched regarding the integrated region as a fixed spatial scope, considering the price method, trade law, and production method [12,23,24,25], or the comprehensive indicator system [26,27], to measure the integration level. This not only has a large subjectivity in selecting relevant indicators but also ignores the spatial dynamic evolution of integrated regions. In reality, regional integration expansion is a common phenomenon, such as the expansion of urban agglomeration in China and the EU [3,20]. Therefore, it is still of great practical significance to consider the impact of regional integration on carbon emissions, considering regional integration expansion. (2) Studies considering the regional integration expansion have gradually increased, but they mainly consider a certain expansion as a quasi-natural experiment [3], and focus on assessing the impact on economic growth [28], industrial structure [29], and environmental quality [18,21]. In general, studies have paid less attention to the carbon emission effect, and the analysis of the heterogeneity effects requires further investigation. (3) The regional integration effect results from complex mechanisms, and the scientific identification of the relevant mechanisms is important in guiding regional integration development. However, available research is relatively insufficient for identifying the complex mechanisms of regional integration that affect economic development [3]. Therefore, there is practical significance in scientifically exploring the carbon reduction effect of regional integration and identifying the complex mechanisms.

For the well-functioning government in China, the government-led urban agglomeration expansion is important to promote regional integration development. The Yangtze River Delta is an urban agglomeration with a higher economic development level, deeper integration level, and faster spatial expansion in China. The regional integration development in the Yangtze River Delta has become a national strategy; deepening urban coordination and actively exploring the low-carbon development path are the practical requirements for striving to be the pioneer of carbon peak and carbon neutral development. Therefore, based on the difference-in-differences model, this study takes the Yangtze River Delta as the research area. The Yangtze River Delta Urban Economic Coordination Committee oversees a quasi-natural experiment to explore the carbon emission reduction effect and driving mechanisms of regional integration from multiple perspectives. This study can enrich the existing research in the following aspects. Different from the previous studies exploring the effect of integration level, it considers the reality of regional integration expansion, enriching the perspective of integration research. In terms of research content, it explores the carbon emission reduction effect and drivers of regional integration from multiple perspectives. The research method regards regional integration expansion as a quasi-natural experiment. The difference-in-differences model is used to conduct the study, which improves the credibility of the research results.

## 2. Theoretical Analysis of the Driving Mechanism

In the process of factor-driven economic development, factor quality and utilization efficiency directly impact urban development quality. This is also affected by the competitive relationship with other cities. As an important factor influencing urban interaction relationships, regional integration affects the quality of urban development. It does this by optimizing the urban development environment and changing the state of competing factors [18,22]. From the research results, regional integration can improve labor productivity [30], accelerate innovation development [22], and improve environmental quality [3,18]. All of these demonstrate the importance of regional integration in improving urban development quality. For example, regional integration promotes the factors of free flow and strengthens urban cooperation [3,22]. This lays the foundation for integrated cities to gather high-quality factors, improve the factor utilization efficiency, and drive urban development transformation from factor-driven to innovation-driven. This, in turn, leads to significant improvement in the quality of urban development.

**Figure 1:**
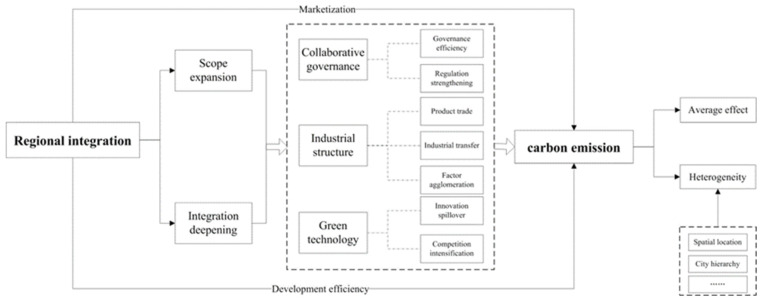
Regional integration and carbon emission linkage mechanism.

From the theme of this research, urban carbon emission is not only related to economic scale. It is also influenced by urban environmental regulation systems, industrial structures, and development paths, which are also significantly driven by competing relationships with other cities [3]. As shown in Figure 1, this study argues that regional integration’s carbon emission reduction effect can be realized through three paths: collaborative governance, industrial structure optimization, and green technology promotion.

### 2.1. Regional Integration Promotes Collaborative Governance

The environment is a public good with typical externalities. As urban economic linkages strengthen and pollution externalities emerge, strengthening collaboration among spatially adjacent cities is an inevitable path to solving regional environmental problems [12]. Due to differences in development levels and resource endowments, heterogeneity exists in the environmental regulation systems of different cities. The disparity in pollution treatment costs distorts the incentives for collaborative treatment, leading some cities to adopt a development model of beggar-thy-neighbor, which is unfavorable for the promotion of environmental collaborative governance and carbon emission reduction [23,31].

Regional integration can reduce urban carbon emissions by promoting collaborative governance. On the one hand, with rapid economic growth driven by regional integration, people’s demand for high-quality environments has increased. Environmental collaborative governance has consequently become an important theme of integration cooperation [3,18]. Environmental regulation measures and relevant policies among cities tend to be consistent, which weakens the incentive distortion due to differences in environmental regulation systems [31]. For example, based on transfer payment for environmental governance cooperation, less-developed cities have obtained sufficient funds to carry out pollution governance. Simultaneously, integrated cities have promoted pollution reduction through improving institutional constraints [17]. On the other hand, different circumstances apply in the regional integration process dominated by developed cities with relatively higher economic development levels. Due to relatively stricter environmental regulations and greater negotiating power, the concentration of high-end elements provides conditions for urban green development and pushes up the green barriers to regional integration development. In this process, the less-developed cities that integrate into the integration region are bound to follow the higher environmental standards set by the developed cities [23]. The improvement of environmental regulations brings about rent-seeking space reduction and pollution costs increase, forcing the shutdown and transfer of high-emission and high-pollution enterprises. These are replaced by clean production methods, thus realizing the reduction of urban carbon emissions [3,31].

### 2.2. Regional Integration Promotes Industrial Structure Optimization

In the industrialization process of China, high carbon emissions are the consequence of an extensive development model with high energy consumption, high pollution, and high emissions. It is caused by the industrial structure directly [4]. In the political promotion game in China featuring economic decentralization and political centralization, local governments choose to adopt the extensive development model of high investment and high energy consumption. This is because of stable economic growth during their tenure. In addition, the competition for growth brings inter-city factor boundary barriers, local protectionism, and a “big and comprehensive” industrial development strategy [3]. This leads to urban development falling into energy dependence and low-end locking dilemmas, inhibiting industrial structure optimization and hindering carbon emission reduction [5,17,32].

Resource mismatch in the segmented market is an important mechanism that inhibits the optimization of industrial structures. Regional integration provides the possibility for urban industrial structure optimization. Firstly, regional integration promotes the accelerated concentration of factors in high-efficiency enterprises and cities. If cities cannot adjust their industrial structure, they will be locked in the value chain’s low end, making it difficult to achieve economic growth and forcing cities to accelerate the industrial structure transformation [3,32]. For example, with the trade facilitation brought by integration, cities with stricter environmental regulations purchase high-emission products from high-efficiency cities. This is done through trade and produces obvious substitution for local high-emission enterprises, presenting two-wheeled development driven by industry transformation and factor matching [5,12,23]. Secondly, the environment is an important input factor for polluting enterprises [23]. According to the classical growth convergence theory, integrated cities have higher factor costs, and residents have a stronger preference for a green environment. This promotes the flow of environmental factors with capital to less-developed regions with higher capital returns. The accelerated migration of high-energy-consuming enterprises reduce carbon emissions in integrated cities. However, other cities accelerate energy consumption and increase carbon emissions while clustering high-energy-consuming enterprises to promote economic development. This reshapes the regional carbon emission pattern [21,31]. Thirdly, regional integration can enhance the urban factor agglomeration level [22]. Developed cities can gather higher-quality factors and achieve lower carbon emissions while promoting industrial structure optimization [3]. In contrast, less-developed cities may become pollution heavens for low-end factors. For example, the controversies over the pollution paradise or pollution halo of foreign direct investment are rooted in the foreign investment quality gap [14,15,31]. Overall, regional integration affects urban carbon emissions through industrial structure optimization and reshapes regional carbon emission patterns.

### 2.3. Regional Integration Accelerates the Green Technology Promotion

Technology promotion is a key factor in promoting high-quality development [22]. The extensive use of low-level technology is an important factor in high carbon emissions in developing countries [3]. This also shows that carbon emissions generated during economic development can be decoupled by applying and promoting carbon-reducing technologies [4,12].

In general, regional integration can enhance green technology promotion and drive carbon emission reduction through the following paths. On the one hand, regional integration brings about the improvement of collaborative innovation platforms and market scale expansion by strengthening urban innovation cooperation, developing the technology trading market, enhancing the innovation spillover effect, and strengthening demand-induced innovation [22]. This can effectively promote the application of advanced technologies and the continuous generation of new technologies. Examples are the diffusion of advanced (or clean) technologies and knowledge that are materialized in products, which provides the possibility for integrated cities to achieve imitative innovation [12,19]. On the other hand, enterprises are the main channel of carbon emissions and innovation development. In a segmented market, enterprises establish their monopoly positions by strengthening links with the government. They lack the incentives to increase R&D investment and progress slowly in implementing production technology, especially low-carbon environmental technology [17]. Deepening regional integration brings increased competition. In order to maintain their dominant position in market competition and occupy a greater market share, enterprises have to increase R&D investment to promote the improvement of production processes and upgrading of products. This will, in turn, drive urban green technology to rise and carbon emissions to decline while enhancing enterprises’ green technology and production efficiency [3,12,19].

Driven by the complex mechanism, costs and benefits in regional integration are also influenced by urban development status. For example, the higher the marketization level, the stronger the ability to gather high-quality factors, which strengthens the positive effects of regional integration. In the process of factor-driven economic development, development efficiency directly influences urban economic growth and carbon emissions. It shows that the more efficient the development and the factor allocation, the lower the carbon emissions. Generally, the marketization level and development efficiency, as characterizations of urban development status and the influential factors of carbon emissions, both regulate the carbon emission effect of regional integration from internal and external perspectives [3,26,31]. In addition, the location conditions and urban hierarchy as exogenous factors also lead to uneven regional integration effects. In the double core-edge process of gradual regional integration expansion, under the mechanism of proximity and hierarchy of spatial spillover effects dominated by developed cities, the benefits of different cities’ participation in regional integration are influenced by development level, spatial location, and other factors. Conversely, under the Matthew effect mechanism of factor concentration and distribution, the differential evolution of urban industrial structure and technology progress also brings about the heterogeneity of regional integration carbon emission effects. Therefore, it is necessary to focus on the moderating effect of city development status and conduct a multi-perspective heterogeneity test.

## 3. Study Area, Model, and Data Explanation

### 3.1. Study Area and Regional Integration

As the economic takeoff is significantly later than that of developed countries, the economic rise of developing countries represented by China has brought about rapid growth in global carbon emissions. For example, according to the Global Carbon Project, as the largest developing country, China’s carbon emissions will reach 11.47 billion tons in 2021, twice that of the United States and four times that of the EU. How to effectively reduce the carbon emissions of developing countries has an especially important impact on global carbon emission reduction. Urban agglomeration, an important carrier for China to participate in global competition, is also important for promoting regional integration and narrowing the regional development gap [22]. Considering the significant spatial correlation of carbon emissions, taking urban agglomeration as a carrier to promote carbon emission reduction has also become the key path to promote China’s green development.

**Figure 2:**
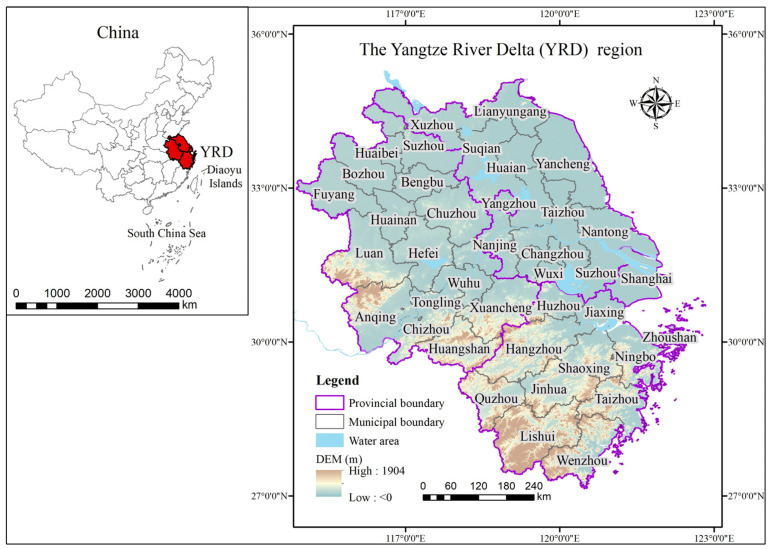
The Yangtze River Delta and administrative divisions.

The Yangtze River Delta urban agglomeration is located on the eastern coast of China. With the promotion of multiple subjects such as government, academia, and enterprises, the cooperation mechanism has been improved, driving the administrative economy to transform into an integrated economy [3]. On 5 November 2018, at the opening ceremony of the “First China International Import Expo” held in Shanghai, China, Xi Jinping, the Chinese president, clearly proposed to support the regional integration development of the Yangtze River Delta and elevate it to a national strategy. This further highlights its strategic status and puts forward higher requirements for urban agglomeration development. The “Outline of the Yangtze River Delta Regional Integrated Development Plan”, issued by the Central Committee of the Communist Party and the State Council of China in 2019, specifies that the regional integration scope of the Yangtze River Delta is the whole area of Shanghai, Jiangsu Province, Zhejiang Province, and Anhui Province (Figure 2). This is also an important basis for the selection of the research scope of this paper. As the sixth-largest urban agglomeration worldwide, the Yangtze River Delta has the most active economic development, the highest degree of openness, and the strongest development strength in China. In 2017, the Yangtze River Delta, with a GDP of CNY 19.53 trillion and a resident population of 224 million, generated 23.61% of GDP, carried 16.08% of the population, and produced 16.56% of carbon emissions on about 3.74% of China’s territory. As China’s economy moves toward a high-quality development stage, whether the Yangtze River Delta can achieve synergy between economy and environment is not only a matter of its own competitiveness. It is also a practical need to explore feasible green development paths for other urban agglomerations and improve the quality of Chinese economic development.

From the existing research [3,31], regional integration and regional integration expansion are two different concepts, although they often accompany each other. That is, the regional integration focuses on the integration level by city cooperation and the integration area is fixed, while regional integration expansion focuses on city cooperation based on the space expansion. For China, the well-functioning government plays a key role in promoting the regional integration development [22]. Since the reform and opening up, China’s economic system reform has greatly stimulated local enthusiasm for economic development. It has also brought many disadvantages, such as beggar-thy-neighbor and homogeneous competition. The State Council established the “Shanghai Economic Zone” to solve this problem. It was an early attempt to promote regional integration development at the national strategic level. Since then, the “Shanghai Economic Zone” has undergone rapid expansion, but because of the lack of administrative authority and difficulties in coordinating regional interests, this attempt to integrate across multiple provincial administrative units failed, and was abolished in 1998. However, the regional integration development of the Yangtze River Delta did not stagnate. In the early 1990s, a series of national strategies, such as the Pudong Development and Opening Up Strategy, the Yangtze River Delta Urban Collaboration Department Directors’ Joint Conference, and the Yangtze River Delta Urban Economic Coordination Council, started a new integration exploration and practice by local governments. At the same time, to support the Yangtze River Delta to participate in international cooperation and competition at a higher level, the central government has issued integration policies to guarantee that the Yangtze River Delta actively explores integration system reform and enhances the integration level. In short, in the synergistic process by the central government and local governments, the regional integration of the Yangtze River Delta is gradually deepening.

Scientifically defining the regional integration process in the Yangtze River Delta is the key foundation for conducting this research. Urban agglomeration integration has multidimensional complexity [22]. The government-led regional integration expansion, as an important means to promote the synergistic evolution of the regional expansion and regional integration level, can avoid the subjective bias of single-indicator or multi-indicator integration measurement. It is also an important basis for current integration research [3,28,30]. Given the differences in the dominant leaders, regional integration in the Yangtze River Delta can be divided into two categories (Table 1). Specifically, central government guidance is based on macro-planning, while local government promotion relies on the Yangtze River Delta Urban Economic Coordination Council (hereinafter referred to as the Coordination Council). For the former, there is a discontinuous dislocation evolution of the urban agglomeration scope. This means that it is affected by the policy withdrawal of some cities. In contrast, the Coordination Council covers the entire area through stepwise expansion, providing a better perspective for this study [22,30]. The Coordination Council, as an official cross-regional cooperation organization, was a response to the central government’s macro policy and the main institutions responsible for economic development. It brings about deepening of the integration level through regular consultation mechanisms among local governments. It also expands its scope by attracting new cities to join, which can better reflect the evolutionary essence of integration in the Yangtze River Delta [30].

This study focuses on whether regional integration can reduce urban carbon emissions in the Yangtze River Delta. Exploring the regional integration effect, it is not only necessary to focus on the impact of regional integration deepening, but also to consider the reality of regional integration expansion [3,30]. Regional integration deepening is the key connotation of regional integration. Whether regional integration expansion can bring about regional integration deepening is the key basis of this study. For the Yangtze River Delta, based on the consultation and cooperation mechanism of the Coordination Council, having a city join the Coordination Council is also an important initiative to strengthen cooperation levels, meaning a synthetic reflection of the reality of the evolution of urban agglomeration regional integration from the perspective of regional integration expansion and regional integration level synergies [22]. Studies have found that in the integration process led by the well-functioning government in China, regional integration expansion is an important measure to promote the deepening of urban agglomeration integration [3,22]. Therefore, the regional integration defined in this study is rationality.

### 3.2. Empirical Models

This study aims to scientifically assess the carbon emission effects of regional integration. However, comparing city carbon emissions before and after participation in the Coordination Council may incorporate other factors into integration shocks. Similarly, comparing the carbon emissions of cities within and outside the Coordination Council will lead to the attribution of unobservable individual systematic variation to the effect. This study uses a difference-in-differences model to obtain an unbiased estimate of the carbon emission effect [3,33]. This method is more mature in terms of causality identification, endogenous treatment, and sample selection bias. It effectively quantifies the policy implementation net effect by controlling the effects of unobservable factors between samples [33]. The benchmark econometric model was constructed as follow:

(1) $$Car_{it}=\alpha_{0}+\alpha_{1}D_{it}+\alpha_{2}X_{it}+\mu_{i}+\eta_{t}+\varepsilon_{it}$$

where *Car* represents urban carbon emissions as the explained variable, α is the regression coefficient, *D* is the explanatory variable, *X* is the control variable, *i* and *t* represent city and time, respectively, *ε* is the random disturbance term, and *μ*_i_ and *η*_t_ are the fixed effects of city and time, respectively.

**Figure 3:**
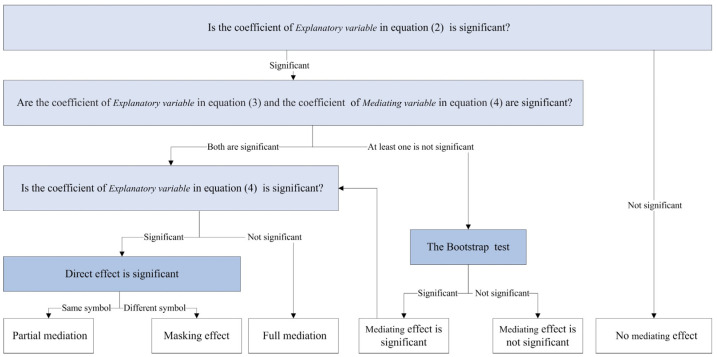
Steps for the mediating effect tests.

The mediating effect models were constructed to identify the mechanisms by which regional integration affects urban carbon emissions [3,22].

(2) $$Car_{it}=\alpha_{0}+\alpha_{1}D_{it}+\alpha_{2}X_{it}+\mu_{i}+\eta_{t}+\varepsilon_{it}$$

(3) $$Med_{it}=\beta_{0}+\beta_{1}D_{it}+\beta_{2}X_{it}+\mu_{i}+\eta_{t}+\varepsilon_{it}$$

(4) $$Car_{it}=\gamma_{0}+\gamma_{1}D_{it}+\gamma_{2}Med_{it}+\gamma_{3}X_{it}+\mu_{i}+\eta_{t}+\varepsilon_{it}$$

where *Med* is the mediating variable, and the remaining variables have the same meaning as in Equation (1). The steps of the mediating effect test were shown in Figure 3. The first step is to test the regression coefficient $\alpha_{1}$ in Equation (2); if significant, the mediating effect will hold, and the subsequent test will be conducted. The second step tests coefficient $\beta_{1}$ in Equation (3) and coefficient $\gamma_{2}$ in Equation (4) in turn, and if both are significant, meaning that the indirect effect is significant, the fourth step test is conducted. If at least one is not significant, a third-step test is performed. The third step uses the bootstrap method to directly test the original hypothesis: $\beta_{1}$ × $\gamma_{2}$ = 0. If significant, the indirect effect holds, and the fourth step is performed. Otherwise, stop the analysis. The fourth step tests the coefficient $\gamma_{1}$ in Equation (4); if not significant, the direct effect is not significant, indicating only a mediating effect. If significant, then the next test is required. The fifth step compares the signs of $\beta_{1}$ × $\gamma_{2}$ and $\gamma_{1}$; if the sign is consistent, a partial mediating effect exists; if the sign is different, there is a masking effect.

The theoretical analysis suggests that other factors also influence the carbon emission effect of regional integration. Drawing on existing studies [22], the moderating effect model is shown in Equation (5).

(5) $$Car_{it}=\varphi_{0}+\varphi_{1}D_{it}+\varphi_{2}Reg_{it}+\varphi_{3}D_{it}\cdot Reg_{it}+\varphi_{}X_{it}+\lambda_{i}+\eta_{t}+\varepsilon_{it}$$

where *Reg* is the moderating variable, and the remaining variables are defined in the same way as in Equation (1).

### 3.3. Variable Description and Data Specification

(1) Explained variables. In view of the practice of carbon peak and carbon neutrality in China, this paper uses carbon emissions as the explained variable. The unit for the variable is million tons.
(2) Explanatory variables. The regional integration in this paper contains the double meaning of integration level deepening and regional integration expansion. Drawing on existing studies [3,33], we construct a dummy variable named integration into the Coordination Council as the explanatory variable: whether city *i* joins the Coordination Council in year *t* is set to 1 if it does; otherwise, it is set to 0.
(3) Control variables. Economic growth is an important source of carbon emissions [3], and GDP is used to characterize economic development (*GDP*); its unit is CNY 1 billion. Government has an important influence on economic development, and government policy regulation cannot be made without the support of fiscal expenditure. We used fiscal expenditure as the proxy variable for local government influence (*Fis*), which is CNY 1 billion [22]. Opening to the outside world influences urban carbon emissions through factor agglomeration, industrial transformation, and innovation evolution [3]. Total imports and exports in billions of yuan characterize it (*Ope*). Infrastructure influences carbon emissions by driving public facility sharing and reducing trade costs, and transportation plays an important role in regional integration [12,31]. The accessibility of transportation (*Tra*) is characterized by passenger volume in a million passengers.
(4) Mediating variables. This study argues that regional integration impacts carbon emissions through three main paths. Collaborative governance indicator (*Cop*), based on the number of signed agreements between governments related to environmental governance, uses the measure of centrality of local government cooperation [34]. This means that the centrality of a city is the sum of other cities and their associated policies, reflecting the active degree of participation in cooperation. The specific method is Equation (6). Industrial structure optimization index (*Str*) is measured by the ratio of the added value of the tertiary industry to the secondary industry. Green technology is an important measure to reduce carbon emissions, and scholars also put forward diversified methods to measure technology level [35,36]. Patent data is often used as a measure of technology innovation because data on the results of the inventive process is highly available, although this data has obvious shortcomings [36]. Considering the importance of technology innovation output in carbon emission reduction and availability of data, the green technology promotion index (*Pat*) was measured by the number of green invention patents granted per 10,000 people.
  (6) $$C(i)=\sum_{j=1,j\neq i}^{N}w_{ij}$$
  where *i* and *j* represent the city serial numbers, *C*(*i*) is the centrality of city *i*, *N* is the number of city units, and *w*_ij_ is the actual amount of connection between city *i* and city *j*.
(5) Moderating Variables. In the process of factor-driven economic development, the marketization level and urban development efficiency are important factors affecting the carbon emission effect of regional integration. The marketization level (*Mark*) characterizes the integration and competitiveness features, which are measured using Wang et al. [37]’s methodology. Urban development efficiency (*EFF*) characterizes factor allocation efficiency and reduces carbon emissions by reducing energy consumption. This is measured by urban green total factor productivity [38].

As cities are the most active areas for economic and social activities, they are the core carriers of regional development and the main source of carbon emissions. Therefore, studying at this scale is more relevant. The study period is 1997–2017. The carbon emission data were obtained from County-level CO_2_ emissions and sequestration databases [1]. Economic and social data were obtained from statistical yearbooks, and the Chinese Research Data Services Platform. Based on the LLC test and ADF Fisher test, the variables are stable sequences. This study is based on the administrative division in the year 2000 to ensure the consistency of sample data. In general, there are 42 cities as the study subjects. It should be noted that more districts and counties in Chaohu City were incorporated into Hefei City and Ma’anshan City. We set that Chaohu City joined the Coordination Council in 2010. In this study, the logarithm of the variables except for explanatory variables was included to enhance the results’ robustness. The summary of the statistics is shown in Table 2.

## 4. Empirical Results

### 4.1. Benchmark Model Results

Table 3 reports the benchmark model regression results based on Equation (1). The regression coefficients of the explanatory variables are all significantly negative when the control variables are partially added in Column (1), or the control variables are all added in Column (2). This indicates that regional integration can significantly reduce urban carbon emissions. In other words, the synergistic evolution of regional integration deepening and regional integration expansion in the Yangtze River Delta contributes to urban green development. In general, government-led regional integration is beneficial to urban carbon emission reduction. It further suggests that promoting carbon peak and carbon neutrality is theoretically feasible based on urban agglomeration integration.

Column (2) estimates the average effect. However, the benefits of cities joining regional integration may vary because of differences in development levels and macro policies [30,39]. Given that carbon emissions are directly influenced by economic scale, this study constructs an interaction term by explanatory variable and GDP. This is incorporated into the benchmark model of Equation (1) to explore the differences effects in cities with different economic scales in Column (3). The coefficient of the explanatory variable is significantly negative, and the coefficient of the interaction term is significantly positive, indicating that the larger the economic scale, the higher the carbon emissions. The threshold value for the effect changing from negative to positive is approximately the size of the city’s GDP, reaching CNY 491.477 billion. In addition, most cities did not exceed this threshold value at the beginning of joining the Coordination Council, confirming regional integration’s carbon emission reduction effect. However, as cities’ GDP grows and crosses this threshold value, regional integration may increase carbon emissions. This study argues that it is driven mainly by the following factors: Economic growth directly influences urban carbon emissions, and the larger the GDP, the larger the carbon emissions. This is mainly in the context of extensive economic growth. A larger GDP characterizes the central position in regional economic growth and factor concentration. The decrease in scale returns and congestion effects caused by excessive factor concentration are important factors leading to an increase in carbon emissions. Therefore, the negative effects of excessive factor concentration should be emphasized in the process of regional integration. That is, optimizing factor allocation in the regional integration is important for urban agglomeration green development.

As a gradual process of regional integration, the Coordination Council promotes a progressive, deepening trend of government cooperation. It characterizes exploration-integration deepening by holding annual joint meetings with mayors [22]. What is the evolutionary pattern of the carbon reduction effect in the process of the gradual expansion and deepening of regional integration? Considering that the Coordination Council was established in 1997, this study measures the effects in the first, second, and third seven-year periods in Column (4). It was found that the coefficients of the explanatory variables are significantly negative at the 1% level, and the longer a city is a member of the Coordination Council, the more obvious the carbon emission reduction effect will be. This also confirms the reality that the regional integration level is gradually deepening while membership in the Coordination Council is increasing. In fact, regional integration based on the game of local interests requires complex negotiations and consultations among cities. The cooperation topic evolution of the Coordination Council also confirms this conclusion. From the first clear proposal of ecological environmental protection in 2003 to ecological environmental governance in 2013 and ecological civilization construction in 2015, the level of environmental collaborative governance has deepened. In addition, the increase in integrated cities is also a foundation for cooperation. This also shows that the deepening of regional integration plays an important role in carbon emissions reduction, which points to the direction of integrated green development.

### 4.2. Robustness Tests

Are the results of benchmark model regressions strongly robust? Based on the differentiation perspectives, the tests in this section verify the robustness of the benchmark model regression results.

#### 4.2.1. Parallel Trend Test

The parallel trend test is a prerequisite for the application of the difference-in-differences model, which requires that the treatment group and the control group have basically the same change trend before the implementation of regional integration [30]. Parallel trend tests have two types of equivalent methods: regression and plotting. The regression method has become a common method [22,33]. In this study, six dummy variables, namely, *post*^−3^, *post*^−2^, *post*^−1^, *post*^0^, *post*^1^, and *post*^2^, were constructed to represent the third year, the second year, and the first year before joining the Coordination Committee, the year of joining the Coordination Committee, and the first year and the second year after joining the Coordination Committee. Then, interaction terms were formed with the dummy variables of the experimental group, namely, *D* × *post*^−3^, *D* × *post*^−2^, *D* × *post*^−1^, *D* × *post*^0^, *D* × *post*^1^, and *D* × *post*^2^. Finally, the explanatory variables in Equation (1) were replaced with the six interaction terms. The results in Table 4 show that the coefficients of *D* × *post*^−3^, *D* × *post*^−2^, and *D* × *post*^−1^ are not significant. This indicates no significant gap in the evolution of carbon emissions between the experimental and control groups before joining the Coordination Council. In other words, the data in this study passed the parallel trend test. In addition, the coefficients of *D* × *post*^0^, *D* × *post*^1^, and *D* × *post*^2^ are all significantly negative and the effects show an expanding trend, which further confirms the conclusions in Table 3. Overall, the data are suitable for the difference-in-differences model, and the estimation results of the benchmark model have strong credibility.

#### 4.2.2. Government Self-Selected Endogeneity Control

The empirical analysis aims to investigate whether regional integration can bring about the carbon emission reduction. The effect assessment based on the difference-in-differences model requires the experimental group selection to be close to random. However, regional integration expansion in the Yangtze River Delta results from local government application and review by the Coordination Council. In fact, the city development level is a key factor in determining whether it can join the Coordination Council. Therefore, government self-selection may result in sample selection bias and affect the regression result robustness in Table 3 [22]. In this study, we use the Heckman two-step method, which constructs the inverse Mills ratio (*IMR*) to control the endogeneity of local government participation in the Coordination Council to verify the robustness of the benchmark model [40]. Column (1) in Table 5 reports the estimation results by controlling the government self-selected factor. The coefficient of IMR is 0.131 and passes the 1% significance level test, suggesting that the sample selection bias does exist, and the benchmark regression model of Column (2) in Table 3 is biased. However, after controlling local government self-selection’s endogeneity in Column (1) in Table 5, the effect remained significantly negative. This verifies the robustness of the benchmark model results.

#### 4.2.3. Controlling Other External Shocks

In terms of macro policy, the central government selects typical cities to carry out pilot projects, which is an important initiative to promote low-carbon transition in China. Studies have found that the low-carbon pilot cities can significantly influence urban carbon emissions [4]. During the study period, the National Development and Reform Commission approved three batches of low-carbon pilot cities, including some cities in the Yangtze River Delta. Does this have a significant impact on benchmark model results? After controlling the external shock of the carbon pilot (*CP*), the coefficient of the explanatory variable is still significantly negative in Column (2) in Table 5. This indicates that the regression results of the benchmark model are strongly robust.

#### 4.2.4. Re-Estimation of Carbon Emission

Carbon emissions estimation directly impacts the results, but it is also a current challenge for relevant research. Drawing on relevant studies [41], the Yangtze River Delta’s carbon emissions data from 1997–2017 were re-estimated. Compared to the estimation of the benchmark model, this method shows obvious differences in the data and methods. The estimation result of the explained variables based on the differentiated method in Column (3) in Table 5 shows that the explanatory variable coefficient is still significantly negative. This again indicates the strong robustness of the benchmark estimation results.

### 4.3. Heterogeneity Tests

In the process of responding to national environmental protection policies and integrating into urban agglomeration, there are significantly different effects due to differences in location conditions, development levels, and city hierarchy [3,24]. For example, studies have found that differences in factor agglomeration capacity because of development level result in heterogeneity in economic growth effects [30,32]. The heterogeneous study can better support the formulation and implementation of relevant policies.

#### 4.3.1. Regional Heterogeneity

Scholars have found that, in the integration development led by developed cities, proximity to developed cities is one of the key factors influencing the effect [22]. The question arises whether the Yangtze River Delta, with Shanghai as its core, has significant differences in the carbon emission effects of different regional cities. In this regard, this study measured the carbon emission effects of regional integration on cities in Jiangsu, Zhejiang, and Anhui. The results are shown in Table 6 in Column (1) to Column (3). The Column (1) is for Jiangsu, Column (2) for Zhejiang, and Column (3) for Anhui.

This study found that the carbon emission effects of cities in different provinces participating in regional integration differed. There was a significant positive effect in Jiangsu, a significant negative effect in Zhejiang, and insignificant negative effect in Anhui. The main reasons for this disparity are as follows.

(1) The manufacturing industry is generally larger in Jiangsu city, and manufacturing is an important source of carbon emissions. When joining the Coordination Council, the rapid agglomeration of manufacturing-related elements increases urban carbon emissions. Jiangsu city benefits from the integrated transportation facilities closely connected to Shanghai, the strong complementarity of industrial innovation in Shanghai, and the superior manufacturing development environment.
(2) In contrast to Jiangsu city, the economic growth of Zhejiang city is significantly driven by the service industry. The transfer of manufacturing and other high-energy-consuming industries, together with the concentration of service industries represented by the Internet in the process of regional integration, are important forces driving industrial upgrading. This is coupled with green technology progress driven by the continuous introduction of green development policies. It results in a decrease in carbon emissions under the synergy of structural transformation and technology progress.
(3) Anhui achieves economic growth by improving infrastructure and undertaking industrial transfers, and industrial agglomeration may increase carbon emissions. At the same time, the process of receiving green technology spillover and industrial transfer from Jiangsu, Zhejiang, and Shanghai is accompanied by the outward diffusion of high-energy-consuming industries in integrated cities. This then realizes technology progress and production efficiency improvement. In contrast to industrial agglomeration and efficiency improvement, the development orientation of economic growth and the lack of high-end factor agglomeration ability led to a relatively strong effect of the former and an insignificant effect of carbon emissions.

#### 4.3.2. City Hierarchy Heterogeneity

Differences in economic development levels, technology endowments, and city hierarchy in the factor-driven development process bring about significant gaps in the competitiveness of urban factors. This leads to heterogeneity in the carbon emission effects of regional integration [3]. It has been found that there are obvious differences between city hierarchies in factor aggregation and innovation development. This leads to differences in regional integration of carbon emission effects [21]. In this section, Shanghai, Nanjing, Suzhou, Wuxi, Hangzhou, Ningbo, and Hefei are classified as high-hierarchy cities, and the rest are general-hierarchy cities. This classification serves to further compare the carbon emission effects of regional integration on city hierarchy.

The results in Table 7 show that regional integration can significantly reduce carbon emissions in high-hierarchy cities. It, however, has an insignificant positive effect on general-hierarchy cities. Overall, factor distribution and industrial transfer in the process of regional integration may increase carbon emissions in some cities. This possible negative effect needs to be further studied. The possible influencing mechanisms for this disparity are as follows.

(1) High-hierarchy cities have higher economic development levels, industrial development efficiency, and environmental regulation perfection. These bear the burden of ecological environmental protection demonstrations. In the process of regional integration development, public facilities further reduce city cooperation costs. They support the development’s efficiency improvement and have the technical ability to accelerate green innovation exploration and gather high-quality development elements. In the regional integration, high-hierarchy cities have gathered efficient green elements and accelerated the outward transfer of high-energy-consuming industries, achieving low-carbon development driven by technology and efficiency.
(2) Industrial division and industrial transfer in regional integration is an important form of urban cooperation and an important driver of carbon emission pattern evolution [21]. A differentiated factor distribution is an important factor in the differences in the carbon emission effect of regional integration. It relates to high efficiency, low energy consumption, and the tendency of high-tech industrial links to gather in high-hierarchy cities. Due to economic development and imperfect environmental regulation systems, general-hierarchy cities tend to become places where low-efficiency and high-energy-consuming industries and related links are undertaken. In addition, general-hierarchy cities increase their carbon emissions while gathering relevant factors to promote economic growth.

## 5. Mechanism Analysis

### 5.1. The Mediating Effect Analysis

This section adopts the mediating effect model of Equations (2)–(4) to quantitatively explore whether regional integration can affect carbon emissions through the paths of collaborative governance, industrial structure optimization, and green technology promotion. This empirically tests the theoretical mechanism.

#### 5.1.1. Collaborative Governance Effect

In the process of regional integration in the Yangtze River Delta, it has been necessary to conduct multi-level government consultation. This is done at the annual meeting of the Coordination Council and is the official process for deepening regional environmental governance. The more cooperation agreements there are among cities, the stronger the collaborative governance degree will be. In this regard, based on the keywords of environment, industry, and innovation, which are closely related to carbon emissions, the textual data of intergovernmental cooperation among cities are retrieved from official websites, and the centrality of policy collaboration is calculated with Equation (6) to test whether regional integration can influence carbon emissions by strengthening collaborative governance.

Table 8 presents the results of the mediating effect tests. Based on Figure 3, the first step in Column (1) shows that the coefficient of the explanatory variable is negative and significant at the 1% level, meaning that there is a mediating effect. The results of Column (2) indicate that regional integration can significantly contribute to the collaborative governance deepening. A possible reason is that regional integration led by local governments significantly promotes the convergence of environmental systems and deepens collaborative governance among different cities. This is also the theme of the annual meeting of the Coordination Commission. In the results of Column (3), the coefficient of the mediating variable is negative and significant at the 1% level, indicating that the indirect effect is significant. Simultaneously, the coefficient of the explanatory variable in Column (3) is also significantly negative. This means that the deepening of collaborative governance partially mediates the reduction of urban carbon emissions. That is, joining regional integration and strengthening policy interactions can significantly promote urban green development. However, the weak effect reflects that there is still much room to improve environmental collaborative governance. For this problem, a possible explanation is that collaborative governance needs more costs.

#### 5.1.2. Industrial Structure Effect

For a long time, the efficiency loss caused by the development of low-end and disorderly industries has been one of the direct factors leading to high carbon emissions [3]. The study of this effect commences by noting the differences in carbon emission intensities of different industries and the advancement of the industrialization process [2,6]. In this study, the ratio of the added value of the tertiary industry to that of the secondary industry is used to measure the industrial structure optimization index. The larger the index, the better the industrial structure. We then test whether regional integration can affect carbon emissions by optimizing industrial structure.

Based on Figure 3, the coefficient of the explanatory variables in Column (4) is significantly negative, indicating that regional integration has a mediating effect on carbon emissions. In the second step of the sequential test in Column (5) and Column (6), this study finds that regional integration significantly affects the mediating variable. That is, the clustering of high-end factors and accelerated industrial transfer in the integration process provides the possibility to optimize industrial structures. However, the coefficient of the mediating variable was not significant in Column (6). The bootstrap test rejects the original hypothesis, which means that regional integration can affect carbon emissions by promoting industrial structure optimization. The fourth and fifth tests showed that the signs of $\beta_{1}$ × $\gamma_{2}$ and $\gamma_{1}$ were inconsistent, implying a masking effect of the mediating variable. That is, although regional integration can promote urban industrial structure optimization, it does not significantly reduce carbon emissions. This is because most cities are still in the rapid industrialization stage, and the characteristics of extensive industrial development and high carbon emission intensity are obvious. The results indicate the importance of industrial structure optimization in carbon emission reduction, and indicate that in the process of achieving green development by integrating the integration region, the focus is optimizing the industrial structure and improving development efficiency.

#### 5.1.3. Green Technology Effect

Relying on green technology to achieve clean production is an important initiative to coordinate economic growth and green development [3,21]. This section uses the number of green invention patents granted per 10,000 people in cities as a mediating variable to explore whether regional integration can influence carbon emissions by improving green technology. Considering that the number of green invention patents granted in some cities is zero during the study period, the mediating variable is constructed by “number of patents plus 1” to ensure the validity of the sample.

Based on the Figure 3, the coefficient of the explanatory variables in Column (7) is significantly negative, indicating that regional integration has a mediating effect on carbon emissions. The results in Column (8) show that regional integration can significantly promote urban green technology, mainly because with the accelerated innovation factor flow, enhanced spatial spillover effect, and expanded market scale, the innovation motivation and innovation efficiency of innovation subjects are significantly enhanced. The results in Column (9) show that the coefficient of the mediating variable is negative and significant at the 1% level, indicating that the indirect effect is significant. Meanwhile, the coefficient of the explanatory variable in Column (9) is also significantly negative, indicating that green technology has a partial mediating effect on carbon emission reduction. This implies that the generation and application of green innovation output not only trigger a change in the production paradigm but also encourage enterprises to update their production technology, machinery, and equipment. All of this contributes to the reduction of urban carbon emissions. However, the weak contribution of green technology reflects the phenomenon of low quality and low conversion rates of innovation. That is, enhancing green innovation in regional integration is an important measure to promote green development, but it is also necessary to focus on innovation quality and the synergy between industrial chains and innovation chains.

### 5.2. The Moderating Effect Analysis

Based on the theoretical analysis, this section explores the moderating effect of urban marketization level and development efficiency on the effect of regional integration on carbon emissions. With the moderating model of Equation (5), Table 9 reports the moderating model estimation results.

#### 5.2.1. Marketization Level Effect

There are significant market distortion factors in China [31]. The lower the marketization level, the more significant the resource mismatch and efficiency loss [16]. A question arises as to whether the marketization level significantly affects the carbon emissions effect of regional integration. With Equation (5), when adding the moderating variable and interaction term in Column (1), the coefficient of the interaction term is significantly negative. This indicates that the higher the marketization level, the stronger the carbon emission reduction effect of regional integration. This may be because the higher the marketization level, the higher the rent-seeking cost, the more market competition, the higher production efficiency, and the stronger innovation momentum; reducing carbon emissions by promoting industrial structure optimization, production technology progress, and energy efficiency improvement. This finding also shows the practical significance of promoting the marketization level, that is, strengthening the economic development efficiency and stimulating the innovative vitality of enterprises. These are important supports for achieving high-quality green development.

#### 5.2.2. Development Efficiency Effect

In the process of factor-driven economic growth, development efficiency reduces carbon emissions by reducing energy consumption and influencing factor agglomeration [17]. This, in turn, moderates the carbon reduction effect of regional integration. With Equation (5), after adding the moderating variable and interaction term in Column (2), development efficiency significantly reduces urban carbon emissions. The interaction term is significantly negative, indicating that development efficiency strengthens the carbon reduction effect of regional integration. This may be because higher development efficiency indicates higher input-output efficiency and lower energy consumption. This is an important initiative to promote carbon emission reduction in economic development. In addition, higher development efficiency also attracts more high-quality factors, which further drives the improvement of urban development quality, and also decreases carbon emissions by optimizing the industrial structure and promoting innovation development. This confirms the heterogeneity test results, and shows that the key basis for integrated cities to achieve carbon emission reduction is to improve development efficiency.

## 6. Discussion

Regional integration can effectively reduce urban carbon emissions, which provides theoretical support for China to promote green development relying on urban agglomeration. However, considering the heterogeneity of carbon emission effects of regional integration, relying on regional integration to achieve low-carbon development should not only formulate localized policies but also pay attention to the possible negative effects. Therefore, we propose the following practical issues that deserve further discussion.

### 6.1. Continuing to Promote the Urban Agglomeration Integration

Scholars have proposed promoting carbon emission reductions by promoting regional market integration, and urban agglomeration integration is an important measure to promote market integration [3,19,22]. The report of the 20th National Congress of the Communist Party of China indicates the importance of regional integration in supporting the construction of a modern socialist country, especially based on urban agglomeration and metropolitan areas. As the Chinese economy shifts to a high-quality development stage, this also puts forward higher requirements for the relationship between economic growth and carbon emissions. Different from previous studies, when considering the regional integration expansion reality, this study also finds that regional integration can significantly reduce urban carbon emissions, indicating that the integrated development of urban agglomeration has the double dividend of economic linkage and environmental optimization [26]. This theoretically supports the idea that China should fully develop the spillover effects of urban agglomeration and build a mutually beneficial green development pattern as it promotes the goal of carbon peak and carbon neutrality.

Although regional integration can significantly reduce urban carbon emissions, it is important to pay attention to the heterogeneity. For example, the different effects among different regions and hierarchies show that urban carbon emission management should not be overly dependent on whether they can join the integration region. The key is how to use the positive effect of regional integration to achieve the improvement of development quality, especially to strengthen the cooperation level with developed cities. The focus should be on collaboratively preparing development plans, breaking down administrative boundary barriers, setting unified market access standards, and promoting the factor free flow. In particular, the longer the city is in the Yangtze River Delta Urban Economic Coordination Council, the more significant is the carbon emission reduction effect. It is clear that in relying on urban agglomeration integration to achieve low-carbon development, the focus should be to deepen the cooperation level. In particular, governments should further improve the regional integration benefits by establishing a collaborative and efficient institutional system, and stimulate the impetus for cities to participate in regional cooperation at a deeper level.

In the regional integration led by the well-functioning government, scholars have realized the importance of integration for high-quality development and attracting cities to participate in integration to achieve higher quality development [3,30]. Expansion has become the normal state of the urban agglomeration integration [22]. For different cities, the benefits of participating in urban agglomeration may also be different, which has been confirmed by many studies [22,39]. The empirical research also finds that, in the process of regional integration led by developed cities in the Yangtze River Delta, the differentiated distribution of factors may increase carbon emissions in some marginal cities. From the perspective of urban agglomeration integration, it also indicates that there may be an optimal boundary for the urban agglomeration scope. That is, although the urban agglomeration integration can activate the Chinese market’s scale advantage, the urban agglomeration scope is not as large as possible. For higher-level governments, scientifically delineating the regional scope of urban agglomeration integration and focusing on promoting the coordination of breadth and depth of regional integration is a real issue.

### 6.2. Scientific Coordination of Economic Growth and Carbon Emission Reduction

Economic growth may lead to an increase in carbon emissions [12]. However, the intensification of global warming has caused great concern in the international community [1,5]. As the largest developing country, the scientific promotion of carbon emission reduction is an important part of the construction of a Chinese ecological civilization, which is also a great power’s responsibility toward global carbon reduction. At this critical period when China is actively moving toward common prosperity and promoting carbon peak and carbon neutrality, it is of practical significance to maintain an appropriate rate of economic growth. Facing the global carbon emission reduction responsibility and the rationality of economic growth, scientifically optimizing the development patterns to achieve green development has become a realistic requirement to promote high-quality development in China.

For developing countries during rapid industrialization, the development pattern dominated by high-pollution and high-emission industries is the direct cause of high carbon emissions [4]. In the process of government-led regional integration, the factor free flow is the driving force of industrial structure evolution. This study shows that industrial structure optimization plays an important role in reducing carbon emissions. Therefore, industrial structure optimization has become an important path to achieving green growth. On the one hand, building a reasonable division based on industrial chains, accelerating the elimination of market segmentation, and strengthening industrial collaboration, will be necessary to collaboratively promote high-quality development while simultaneously improving factor efficiency. On the other hand, local governments should always adhere to the concept of green development, based on a strict negative list system. They need to strictly limit the development of high-energy-consuming and high-emission industries, such as cement, iron, steel, and the chemical industry, and encourage the development of high-tech enterprises and strategic emerging industries. For the Yangtze River Delta, it is necessary to promote industrial structure upgrading and achieve high-quality green development by limiting high emission enterprises and encouraging high-tech enterprises [3]. Facing factor competition and unbalanced development, relevant initiatives should be adopted according to urban conditions. For example, cities should focus on specific links based on economic development stages and resource endowments, promoting economic growth and improving cooperation efficiency by eliminating misalignment in industrial chains [19,31]. Special focus should be placed on differentiated and misaligned development between cities, avoiding less-developed cities becoming the sites of pollution undertaking.

Innovation is an important initiative to achieve low-carbon development [3,31]. The empirical research in this study verifies this conclusion, and scholars have verified the importance of regional integration for innovation development [22]. Therefore, promoting technology progress through urban agglomeration integration is an important path to achieve green development [26]. To this end, the Yangtze River Delta should first improve its infrastructure, especially the high-speed railroad network. This will accelerate the radiation of innovation resources on a larger scale. Second, it can give full play to innovation axes’ cascading and radiating role, such as the Shanghai–Nanjing industrial innovation belt. In innovation cooperation based on city endowments, it is also necessary to strengthen the level of innovation division; developed cities should focus on original and frontier technology breakthroughs, and the less-developed cities should collaborate to promote technology progress and green production by actively undertaking and transforming related technologies. However, the low contribution rate of innovation in the mediating effect test indicates that innovation quality improvement is important. That is, we should pay more attention to the industrial application of green innovation technologies. Focused attention must be placed on strengthening cross-regional cooperation based on the synergy of industrial chains. Innovation chains should be used to build an efficient innovation development pattern of government, industry, academia, and research, supporting urban agglomerations to take the lead in achieving carbon peak and carbon neutrality with innovation development.

### 6.3. Deeply Promoting the Reform of the Political Assessment System

In China, the macro policies by the central government, especially the assessment system, have an important influence on local government behavior. Since the reform and opening up, GDP tournaments have led local governments to introduce many high-energy-consuming and high-polluting enterprises in pursuit of political achievements. These have brought serious problems while achieving economic growth [23]. Under the current system of economic decentralization and political centralization in China, the extensive economic growth pattern of local governments, market segmentation among cities, and beggar-thy-neighbor environmental protection all have institutional realities. Therefore, in the process of promoting green development, it is necessary to build a corresponding institutional guarantee system, providing institutional guarantees for promoting green development based on urban agglomeration integration.

Under the vertical management system, the central government has a direct impact on the economic development of local governments [3]. For the central government, the key is to guide local governments to pay attention to environmental protection in economic growth, and turn from pollution before treatment to green development. In the institutional reform of coordinating economic growth and environmental protection, the central government should, firstly, guide local economic behavior through performance assessment. Secondly, it should provide institutional guarantees for regional integration development. By accelerating the reform of the government performance appraisal system and the promotion mechanism of officials, the central government can incorporate carbon emission reduction into the appraisal system. We suggest taking this as a hard indicator. It can also explore an evaluation index system for green development that can be monitored and operated, which would force local governments to shift from simply pursuing economic growth to focusing on development quality. Of course, the relevant system must be established on the basis of scientific analysis. The development and implementation of policies should be adjusted dynamically in keeping with scientific assessment, promoting regional integration as a realistic strategy for green development.

Local government is the main body of economic growth and the important undertaker of carbon emission reduction [5,12]. For local governments, they should accelerate the exploration of feasible institutional innovation, and promote the urban agglomeration green development through strengthening cooperation and promoting the factor flow. The feasible institutional exploration includes: exploring the vertical environmental management model, establishing an environmental governance fund, accelerating the unified environmental standards, and strengthening joint environmental enforcement efforts. The government can also cultivate the environmental technology market, actively conduct third-party environmental monitoring of government procurement, and encourage private capital to enter the pollution control field. During local government cooperation, developed cities have the motivation to formulate policies that are more biased towards their own development, which may also be detrimental to the implementation of relevant policies. Therefore, they also need the guidance of the central government in the formulation of relevant policies to promote high-quality green development.

### 6.4. Limitations

Different to previous studies on the regional integration definition [31,42], this study systematically evaluated the carbon emission effect of regional integration in the Yangtze River Delta, taking into account the reality of the coordinated evolution of regional integration deepening and regional integration expansion. This is of great significance for urban agglomeration integration. Considering the complexity and uncertainty of urban agglomeration systems, this study also has the following shortcomings. The selection of scientific variables is the key basis for empirical research. For the drastic adjustment in city administrative divisions and imperfect statistical data, variables need to be improved in this study. Despite the comparative study of this effect from multiple perspectives, the classification based on scale, location, and hierarchy are still relatively extensive. Limited by data availability and research themes, this study focused on the effects of joining the Yangtze River Delta Urban Economic Coordination Council, but it did not address the effects of specific actions after joining. With the accumulation and improvement of relevant data, these issues are important directions to consider and study in depth.

## 7. Conclusions

Facing the regional integration led by the well-functioning government in China, an increasing number of cities expect to participate in integration to achieve higher-quality development. Expansion has become a common phenomenon of regional integration. Based on city-scale data from 1997 to 2017, this study treats the expansion of the Yangtze River Delta Urban Economic Coordination Council as a quasi-natural experiment. With the difference-in-differences model, we estimate the carbon emission effect of regional integration. It was found that regional integration can significantly reduce urban carbon emissions, which indicates the feasibility of low-carbon development reliant on urban agglomeration integration. The carbon emission effects of regional integration show multiple heterogeneities, for example, the significant negative effect of high-hierarchy cities and the insignificant positive effect of general-hierarchy cities. Driving mechanism tests based on the mediating effect model and the moderating effect model found that regional integration influences urban carbon emissions through collaborative governance, industrial structure optimization, and green technology promotion. However, the carbon emission effect of regional integration is significantly moderated by marketization level and development efficiency. Based on the relevant empirical research, we propose to better promote the high-quality development of China’s economy by strengthening urban agglomeration cooperation, optimizing urban development paths, strengthening innovative development, and improving macro political systems. It should be emphasized that the implementation of relevant policies to promote coordinated development should be formulated after considering local conditions.

## Figures and Tables

**Table 1:** The regional integration scope evolution of the Yangtze River Delta.

Type	Meaningful Event	Regional Integration Scope
Leading by central government	Guiding Opinions on Further Promoting the Reform and Opening up and Economic and Social Development in the Yangtze River Delta in 2008	Shanghai, Nanjing, Suzhou, Wuxi, Changzhou, Zhenjiang, Yangzhou, Taizhou, Nantong, Huai’an, Suqian, Yancheng, Xuzhou, Lianyungang, Hangzhou, Ningbo, Wenzhou, Huzhou, Jiaxing, Shaoxing, Jinhua, Quzhou, Zhoushan, Taizhou, Lishui
	Regional Planning of the Yangtze River Delta in 2010	Shanghai, Nanjing, Suzhou, Wuxi, Changzhou, Zhenjiang, Yangzhou, Taizhou, Nantong, Hangzhou, Ningbo, Huzhou, Jiaxing, Shaoxing, Zhoushan, Taizhou
	Guiding Opinions on Promoting the Development of the Yangtze River Economic Belt by Relying on the Golden Waterway in 2014	Shanghai, Nanjing, Suzhou, Wuxi, Changzhou, Zhenjiang, Yangzhou, Taizhou, Nantong, Huai’an, Suqian, Yancheng, Xuzhou, Lianyungang, Hangzhou, Ningbo, Wenzhou, Huzhou, Jiaxing, Shaoxing, Jinhua, Quzhou, Zhoushan, Taizhou, Lishui, Hefei, Huangshan, Wuhu, Ma’anshan, Anqing, Huainan, Fuyang, Huaibei, Tongling, Bozhou, Xuancheng, Bengbu, Lu’an, Chuzhou, Chizhou, Suzhou
	Development planning of urban agglomeration in the Yangtze River Delta in 2016	Shanghai, Nanjing, Wuxi, Changzhou, Suzhou, Nantong, Yancheng, Yangzhou, Zhenjiang, Taizhou, Hangzhou, Ningbo, Jiaxing, Huzhou, Shaoxing, Jinhua, Zhoushan, Taizhou, Hefei, Wuhu, Ma’anshan, Tongling, Anqing, Chuzhou, Chizhou, Xuancheng
	Outline of the Yangtze River Delta Regional Integrated Development Plan in 2019	Shanghai, Nanjing, Suzhou, Wuxi, Changzhou, Zhenjiang, Yangzhou, Taizhou, Nantong, Huai’an, Suqian, Yancheng, Xuzhou, Lianyungang, Hangzhou, Ningbo, Wenzhou, Huzhou, Jiaxing, Shaoxing, Jinhua, Quzhou, Zhoushan, Taizhou, Lishui, Hefei, Huangshan, Wuhu, Ma’anshan, Anqing, Huainan, Fuyang, Huaibei, Tongling, Bozhou, Xuancheng, Bengbu, Lu’an, Chuzhou, Chizhou, Suzhou
Leading by local government	The 1th Meeting of the Yangtze River Delta Urban Economic Coordination Committee in 1997	Shanghai, Nanjing, Suzhou, Wuxi, Changzhou, Zhenjiang, Yangzhou, Taizhou, Nantong, Hangzhou, Ningbo, Huzhou, Jiaxing, Shaoxing, Zhoushan
	The 4th Meeting of the Yangtze River Delta Urban Economic Coordination Committee in 2003	Shanghai, Nanjing, Suzhou, Wuxi, Changzhou, Zhenjiang, Yangzhou, Taizhou, Nantong, Hangzhou, Ningbo, Huzhou, Jiaxing, Shaoxing, Zhoushan, Taizhou
	The 10th Meeting of Yangtze River Delta Urban Economic Coordination Committee in 2010	Shanghai, Nanjing, Suzhou, Wuxi, Changzhou, Zhenjiang, Yangzhou, Taizhou, Nantong, Hangzhou, Ningbo, Huzhou, Jiaxing, Shaoxing, Zhoushan, Taizhou, Hefei, Yancheng, Ma’anshan, Jinhua, Huai’an, Quzhou
	The 13th Meeting of Yangtze River Delta Urban Economic Coordination Committee in 2013	Shanghai, Nanjing, Suzhou, Wuxi, Changzhou, Zhenjiang, Yangzhou, Taizhou, Nantong, Hangzhou, Ningbo, Huzhou, Jiaxing, Shaoxing, Zhoushan, Taizhou, Hefei, Yancheng, Maanshan, Jinhua, Huai’an, Quzhou, Xuzhou, Wuhu, Chuzhou, Huainan, Lishui, Wenzhou, Suqian, Lianyungang
	The 18th Meeting of Yangtze River Delta Urban Economic Coordination Committee in 2018	Shanghai, Nanjing, Suzhou, Wuxi, Changzhou, Zhenjiang, Yangzhou, Taizhou, Nantong, Hangzhou, Ningbo, Huzhou, Jiaxing, Shaoxing, Zhoushan, Taizhou, Hefei, Yancheng, Maanshan, Jinhua, Huai’an, Quzhou, Xuzhou, Wuhu, Chuzhou, Huainan, Lishui, Wenzhou, Suqian, Lianyungang, Tongling, Anqing, Chizhou, Xuancheng
	The 19th Meeting of Yangtze River Delta Urban Economic Coordination Committee in 2019	Shanghai, Nanjing, Suzhou, Wuxi, Changzhou, Zhenjiang, Yangzhou, Taizhou, Nantong, Hangzhou, Ningbo, Huzhou, Jiaxing, Shaoxing, Zhoushan, Taizhou, Hefei, Yancheng, Maanshan, Jinhua, Huai’an, Quzhou, Xuzhou, Wuhu, Chuzhou, Huainan, Lishui, Wenzhou, Suqian, Lianyungang, Tongling, Anqing, Chizhou, Xuancheng, Bengbu, Huangshan, Lu’an, Huaibei, Suzhou, Bozhou, Fuyang

**Table 2:** Summary of statistics.

Variable	Obs	Mean	SD	Min	Max
*Car*	882	26.52	30.40	1.47	230.71
*D*	882	0.48	0.50	0	1
*GDP*	882	1579.14	2437.36	55.49	23,094.78
*Fis*	882	218.82	547.48	3.35	7064.89
*Ope*	882	984.83	2925.04	0.37	24,265.55
*Tra*	882	13,103.91	11,818.90	448.00	82,948.00
*Cop*	882	5.37	4.56	0	24
*Str*	882	0.85	0.27	0.31	2.34
*Pat*	882	68.82	225.65	0	2481
*Mark*	882	8.83	3.47	0.60	17.00
*EFF*	882	4.60	3.83	0.45	22.47

**Table 3:** Benchmark regression results.

Variable	(1)	(2)	(3)	(4)
*D*	−0.0342 *** (−3.06)	−0.0348 *** (−3.05)	−0.170 *** (−2.62)	
D*GDP			0.0200 ** (2.11)	
*year*_1–7_				−0.0463 *** (−3.67)
*year*_8–14_				−0.0557 *** (−3.12)
*year*_15–21_				−0.112 *** (−4.58)
Control variables	Partially	YES	YES	YES
City/Year FE	YES	YES	YES	YES
Constant	−0.216 (−1.21)	−0.195 (−1.01)	−0.107 (−0.54)	−0.132 (−0.68)
R-squared	0.888	0.883	0.880	0.893
Observations	882	882	882	882

Note: t-values are given in parentheses. ** *p* < 0.05. *** *p* < 0.01.

**Table 4:** Results of the parallel trend test.

Variable	(1)	(2)	(3)	(4)
*D* × *post*^−3^	−0.0270 (−1.31)	−0.0267 (−1.29)	−0.0270 (−1.30)	−0.0273 (−1.32)
*D* × *post*^−2^	−0.0309 (−1.50)	−0.0309 (−1.49)	−0.0323 (−1.35)	−0.0326 (−1.57)
*D* × *post*^−1^	−0.0409 (−1.58)	−0.0408 (−1.59)	−0.0411 (−1.58)	−0.0424 (−1.58)
*D* × *post*^0^		−0.0260 ** (−2.16)	−0.0211 ** (−2.15)	−0.0166 ** (−2.10)
*D* × *post*^1^			−0.0318 *** (−2.75)	−0.0322 *** (−2.77)
*D* × *post*^2^				−0.0410 *** (−3.10)
Control variables	YES	YES	YES	YES
City/Year FE	YES	YES	YES	YES
Constant	0.129 (0.70)	0.127 (0.69)	0.130 (0.71)	−0.189 (−0.99)
R-squared	0.879	0.879	0.879	0.881
Observations	882	882	882	882

Note: t-values are given in parentheses. ** *p* < 0.05. *** *p* < 0.01.

**Table 5:** Results of robustness tests.

Variable	(1)	(2)	(3)
*D*	−0.0669 *** (−3.99)	−0.0352 *** (−3.08)	−0.0360 *** (−2.99)
*IMR*	0.131 *** (12.81)		
*CP*		−0.00869 (−0.64)	
*H-Tra*			
Control variables	YES	YES	YES
City/Year FE	YES	YES	YES
Constant	−0.598 *** (−3.55)	−0.184 (−0.95)	1.400 *** (6.95)
R-squared	0.835	0.884	0.859
Observations	882	882	882

Note: t-values are given in parentheses. *** *p* < 0.01.

**Table 6:** Heterogeneity of effects in different regions.

Variable	(1)	(2)	(3)
*D*	0.0439 ** (2.48)	−0.0405 ** (−2.36)	−0.0266 (−1.56)
Control variables	YES	YES	YES
City/Year FE	YES	YES	YES
Constant	−0.122 (−0.64)	−0.0513 (−0.27)	−0.144 (−0.74)
R-squared	0.882	0.885	0.881
Observations	273	231	357

Note: t-values are given in parentheses. ** *p* < 0.05.

**Table 7:** Heterogeneity of effects in different hierarchies.

Variable	High-Hierarchy Cities		General-Hierarchy Cities	
	(1)	(2)	(3)	(4)
*D*	0.665 *** (3.01)	−0.0775 ** (−2.20)	0.706 *** (12.51)	0.00356 (0.31)
Control variables	NO	YES	NO	YES
City/Year FE	NO	YES	NO	YES
Constant	2.771 *** (74.16)	−0.0320 (−0.17)	2.638 *** (109.39)	−0.0746 (−0.39)
R-squared	0.0149	0.872	0.0175	0.882
Observations	147	147	735	735

Note: t-values are given in parentheses. ** *p* < 0.05. *** *p* < 0.01.

**Table 8:** Regression results of the mediating effect model.

Variable	Collaborative Governance			Industrial Structure			Green Technology		
	(1)	(2)	(3)	(4)	(5)	(6)	(7)	(8)	(9)
*D*	−0.0348 *** (−3.05)	5.012 *** (3.00)	−0.0129 *** (−3.66)	−0.0348 *** (−3.05)	0.0423 ** (2.11)	−0.0355 *** (−3.10)	−0.0348 *** (−3.05)	0.0107 ** (2.13)	−0.0347 *** (−3.04)
*Cop*			−0.00436 *** (−2.86)						
*Str*						0.0165 (0.91)			
*Pat*									−0.00903 * (−1.87)
Control variables	YES	YES	YES	YES	YES	YES	YES	YES	YES
City/Year FE	YES	YES	YES	YES	YES	YES	YES	YES	YES
*Constant*	−0.195 (−1.01)	9.016 * (1.80)	−0.0182 (−0.88)	−0.195 (−1.01)	2.314 *** (6.88)	−0.217 (−1.12)	−0.195 (−1.01)	−18.013 *** (−12.97)	−0.357 * (−1.70)
R-squared	0.883	0.166	0.862	0.883	0.0229	0.882	0.883	0.311	0.888
Observations	882	882	882	882	882	882	882	882	882

Note: t-values are given in parentheses. * *p* < 0.1. ** *p* < 0.05. *** *p* < 0.01.

**Table 9:** Results of the test for adjusting effect.

Variable	(1)	(2)
*D*	−0.0416 *** (−3.72)	−0.0263 ** (−2.26)
*Mark*	0.949 *** (5.07)	
*D* × *Mark*	−0.445 ** (−2.34)	
*Eff*		−0.0187 *** (−4.88)
*D* × *Eff*		−0.0163 *** (−4.53)
Control variables	YES	YES
City/Year FE	YES	YES
Constant	−0.387 ** (−2.04)	−0.942 *** (−3.80)
R-squared	0.858	0.901
Observations	882	882

Note: t-values are given in parentheses. ** *p* < 0.05. *** *p* < 0.01.

## Data Availability

Chinese Research Data Services Platform (http://www.cnrds.com accessed on 6 May 2022); Shanghai Statistical Yearbook (https://tjj.sh.gov.cn/tjnj/index.html accessed on 6 May 2022); Jiangsu Statistical Yearbook (http://tj.jiangsu.gov.cn/col/col83749/index.html accessed on 12 May 2022); Zhejiang Statistical Yearbook (http://tjj.zj.gov.cn/col/col1525563/index.html accessed on 15 May 2022); Anhui Statistical Yearbook (http://tjj.ah.gov.cn/ssah/qwfbjd/tjnj/index.html accessed on 20 May 2022); Carbon Emission Accounts and Datasets for Emerging Economies (https://www.ceads.net.cn/ accessed on 30 May 2022).
